# Metabolic Engineering of *Bacillus amyloliquefaciens* to Efficiently Synthesize L-Ornithine From Inulin

**DOI:** 10.3389/fbioe.2022.905110

**Published:** 2022-06-08

**Authors:** Yifan Zhu, Yi Hu, Yifan Yan, Shanshan Du, Fei Pan, Sha Li, Hong Xu, Zhengshan Luo

**Affiliations:** ^1^ State Key Laboratory of Materials-Oriented Chemical Engineering, Nanjing Tech University, Nanjing, China; ^2^ College of Food Science and Light Industry, Nanjing Tech University, Nanjing, China

**Keywords:** *Bacillus amyloliquefaciens*, L-Ornithine, L-ornithine transporter, non-grain raw materials, modular metabolic engineering

## Abstract

*Bacillus* amyloliquefaciens is the dominant strain used to produce γ-polyglutamic acid from inulin, a non-grain raw material. B. amyloliquefaciens has a highly efficient tricarboxylic acid cycle metabolic flux and glutamate synthesis ability. These features confer great potential for the synthesis of glutamate derivatives. However, it is challenging to efficiently convert high levels of glutamate to a particular glutamate derivative. Here, we conducted a systematic study on the biosynthesis of L-ornithine by *B. amyloliquefaciens* using inulin. First, the polyglutamate synthase gene pgsBCA of *B. amyloliquefaciens* NB was knocked out to hinder polyglutamate synthesis, resulting in the accumulation of intracellular glutamate and ATP. Second, a modular engineering strategy was applied to coordinate the degradation pathway, precursor competition pathway, and L-ornithine synthesis pathway to prompt high levels of intracellular precursor glutamate for l-ornithine synthesis. In addition, the high-efficiency L-ornithine transporter was further screened and overexpressed to reduce the feedback inhibition of L-ornithine on the synthesis pathway. Combining these strategies with further fermentation optimizations, we achieved a final L-ornithine titer of 31.3 g/L from inulin. Overall, these strategies hold great potential for strengthening microbial synthesis of high value-added products derived from glutamate.

## Introduction

With the development of synthetic biology, chemical and material products are increasingly being obtained by microbial synthesis (McCarty and Ledesma-Amaro, 2019; Luo et al., 2021). These products are mainly obtained through microbial fermentation using food materials (glucose, sucrose, and starch) as substrates (Qiu et al., 2017; Jiang et al., 2019). The main reason is that food materials are easy to use, and their metabolic pathways have also been extensively studied. Nonetheless, the demand for various products continues to increase, and microbial fermentation using food materials has some shortcomings, i.e., competition with humans for grain resources (Chen et al., 2020). In recent years, research on the use of non-grain food materials for fermentation has been growing steadily. The use of non-grain food materials can make up for the shortage of raw food materials. Nevertheless, non-grain food materials are difficult to use, warranting the development of methods for efficient processing of these materials. To improve efficiency in the utilization of non-grain raw materials, we need to overcome limitations such as insufficient degrading enzymes and stress factor inhibition. The development of metabolic engineering and synthetic biology provides a means to solve these problems.

L-Ornithine is an essential non-protein amino acid widely used in the food, pharmaceutical, and chemical industries (Sheng et al., 2021). It is an essential part of the urea cycle and is widely used to treat liver diseases and burns. In recent years, the development of L-ornithine products (sweeteners, hepatoprotective drugs, and slimming products) has been gradually increasing, resulting in a rapidly growing demand for L-ornithine (Zajac et al., 2010). At present, L-ornithine obtained by chemical synthesis and enzymatic catalysis cannot meet the increasing demand. In addition, chemical synthesis has some drawbacks: chemically synthesized L-ornithine is a mixture of d-ornithine and l-ornithine (only L-ornithine has biological activity) and cannot be directly used in the pharmaceutical and food industries (Shu et al., 2018a). Fortunately, the rapid development of synthetic biology provides new ways for microorganisms to synthesize L-ornithine. Research on L-ornithine production by biological fermentation thus far has mainly focused on *Corynebacterium glutamicum* and *Escherichia coli*. Hwang et al. overexpressed the l-ornithine synthesis pathway genes (*argCJBD*) in an engineered strain of *C. glutamicum* (*C.glu/ΔargF/ΔargR/Δprob*), and the level of L-ornithine fermentation increased by 30% to 16.49 g/L (Hwang et al., 2008). Lee et al. overexpressed N-acetyl synthase (ArgA214) in *E. coli* and knocked out speF and proB, and the L-ornithine titer reached 13.2 g/L (Lee and Cho, 2006). Current research on the biosynthesis of L-ornithine mainly uses food materials for fermentation. With the increased demand for L-ornithine in the pharmaceutical and food industries, the production scale will inevitably need to expand. This will require more food materials for fermentation. Moreover, an insufficient supply of food materials for fermentation will undoubtedly become a significant problem.

Jerusalem artichoke, a non-grain food crop, is considered a potential energy source because of its high sugar content. Jerusalem artichoke contains the storage polysaccharide, inulin, which accounts for 85% of its dry weight (Li et al., 2014). This polysaccharide can be used with simple preprocessing. In recent years, Jerusalem artichoke has been used as substrate to produce products such as ethanol (Zhang et al., 2010; Lim et al., 2011), lactic acid (Ge et al., 2009), and 2,3-butanediol (Sun et al., 2009). In previous studies, we isolated and identified a bacterial strain from the root of Jerusalem artichoke, named *B. amyloliquefaciens* NB (Qiu et al., 2019b). This strain is deposited in the China Center for Type Culture Collection (China Center for Type Culture Collection), and the deposit number is CCTCC NO: M2016346*.* This strain can efficiently use the crude extract of Jerusalem artichoke (non-grain food material). Furthermore, *B. amyloliquefaciens* NB can efficiently synthesize poly-γ-glutamic acid (γ-PGA) without exogenous addition of glutamine (Sha et al., 2020a). Since γ-PGA is a glutamate polymer, *B. amyloliquefaciens* NB might have a highly efficient tricarboxylic acid cycle metabolic flux and glutamate synthesis ability. These characteristics highlight the potential for synthesizing glutamate derivatives from non-grain raw materials in *B. amyloliquefaciens* through the rational design of metabolic pathways.

In the present study, *B. amyloliquefaciens* NB was engineered for *de novo* biosynthesis of L-ornithine from inulin. Upon knocking out the γ-PGA synthase gene in *B. amyloliquefaciens* NB, intracellular glutamate and ATP levels were enhanced by 7and 4.5-fold, respectively. The glutamate degradation pathway, the precursor competition pathway, the L-ornithine synthesis pathway, and the L-ornithine transport process were further coordinated to prompt the high level of intracellular precursor glutamate into L-ornithine synthesis. Building upon these results, we successfully achieved a final titer of 31.3 g/L L-ornithine from non-food raw materials (inulin) in an optimized bioreactor system. Overall, this study aimed to provide a reference for improving L-ornithine synthesis and a strategy for the use of non-grain raw materials for microbial synthesis of value-added products derived from glutamate.

## Materials and Methods

### Microorganisms and Plasmids

The bacterial strains and main plasmids used in this study are listed in Table 1. *Escherichia coli* DH5α was used as a recipient for plasmid construction. We used the dam^−^ and dcm^−^-deficient host *E. coli* GM2163 to prepare the unmethylated plasmids (Sha et al., 2020a).

**TABLE 1:** Strains and plasmids used in this study.

Strains or plasmids	Relevant properties	Source
*E. coli* DH5α	F^−^, φ80d*lac*Z△M1, △(*lacZYA*-*argF*) U169, *deoR*, *recA*1, *endA1*, *hsdR*17 (rk^−^, mk^+^), *phoA*, *supE*44, λ^−^ *thi*-1, *gyrA*96, *relA*1	
*E. coli* GM2163	F^−^, *ara*-14 *leuB*6 *thi*-1 *fhu*A31 *lacY*1 *tsx*-78 *galK*2 *galT*22 *supE*44 *hisG*4 *rpsL* 136 (*Str* ^*R*^) *xyl*-5 *mtl*-1 *dam*13::Tn9 (*Cam* ^*R*^) *dcm*-6 *mcrB*1 *hsdR*2 *mcrA*	Qiu et al. (2017)
*B. amyloliquefaciens* NB	Glutamate-independent *B. amyloliquefaciens* can produce high yield of polyglutamic acid	Qiu et al. (2017)
*B. amyloliquefaciens* NB(Δ*pgsBCA*)	*B. amyloliquefaciens NB* derivate, deletion of *pgsBCA*	Sha et al. (2020a)
*B. amyloliquefaciens* NB(Δ*pgsA*)	*B. amyloliquefaciens NB* derivate, deletion of *pgsA*	This study
*B. amyloliquefaciens* NB(Δ*pgsB*)	*B. amyloliquefaciens NB* derivate, deletion of *pgsB*	This study
*B. amyloliquefaciens* NB(Δ*pgsC*)	*B. amyloliquefaciens NB* derivate, deletion of *pgsC*	This study
*B. amyloliquefaciens* NBO1	NB*ΔpgsBCA* derivate, deletion of *argF*	This study
*B. amyloliquefaciens* NBO2	NB*ΔpgsBCA* derivate, deletion of *argI*	This study
*B. amyloliquefaciens* NBO3	NBO1 derivate, deletion of *argI*	This study
*B. amyloliquefaciens* NBO4	NBO3 derivate, deletion of *speF*	This study
*B. amyloliquefaciens* NBO5	NBO3 derivate, deletion of *prob*	This study
*B. amyloliquefaciens* NBO6	NBO3 derivate, deletion of *speF* gene and *prob* gene	This study
*B. amyloliquefaciens* NBO7	NBO6 derivate, carrying pHY-*ArgA*	This study
*B. amyloliquefaciens* NBO8	NBO6 derivate, carrying pHY-*ArgB*	This study
*B. amyloliquefaciens* NBO9	NBO6 derivate, carrying pHY-*ArgC*	This study
*B. amyloliquefaciens* NBO10	NBO6 derivate, carrying pHY-*ArgD*	This study
*B. amyloliquefaciens* NBO11	NBO6 derivate, carrying pHY-*ArgE*	This study
*B. amyloliquefaciens* NBO12	NBO6 derivate, carrying pHY-*ArgA*-*ArgE*	This study
*B. amyloliquefaciens* NBO13	NBO6 derivate, carrying pHY-*ArgABCDE*	This study
*B. amyloliquefaciens* NBO14	NBO6 derivate, carrying pHY- *Cg.lysE*	This study
*B. amyloliquefaciens* NBO15	NBO6 derivate, carrying pHY- *E.coli.lysE*	This study
*B. amyloliquefaciens* NBO16	NBO6 derivate, carrying pHY- *BS.lysE*	This study
*B. amyloliquefaciens* NBO17	NBO6 derivate, carrying pHY- *BA.lysE*	This study
*B. amyloliquefaciens* NBO18	NBO12 derivate, overexpression of *BA.lysE*	This study
Plasmids		
pHT-01	*Amp* ^*r*^, *Cm* ^*r*^, *E. coli*−*B. subtilis* shuttle vector	Qiu et al. (2020a)
pDR244	*Amp* ^*r*^, *Spec* ^*r*^, cre/lox-mediated *E. coli*-*Bacillus* shuttle vector	Qiu et al. (2020a)
pHY	*Amp* ^*r*^, *Cm* ^*r*^, *E. coli*−*B. subtilis* shuttle vector, containing the constitutive strong promoter *P*_*HpaII*_, p15A ori	Qiu et al. (2020a)
pHT-Cas9n	pHT derivate consists of the promoter *P*_*grac*_, Cas9n and the terminator *T*_*amy*_	Qiu et al. (2020a)
pDR-*pgssgRNA*	pDR derivate consists of the promoter *P*_*HpaⅡ*_, *sgRNA*, *Tamy*, the up and downstream fragment of *pgsBCA* gene	This study
pDR-*argIsgRNA*	pDR derivate consists of the promoter *P*_*HpaⅡ*_, *sgRNA*, *Tamy*, the up and downstream fragment of *argI* gene	This study
pDR-*argFsgRNA*	pDR derivate consists of the promoter *P*_*HpaⅡ*_, *sgRNA*, *Tamy*, the up and downstream fragment of *argF* gene	This study
pDR-*speFsgRNA*	pDR derivate consists of the promoter *P*_*HpaⅡ*_, *sgRNA*, *Tamy*, the up and downstream fragment of *speF* gene	This study
pDR-*probsgRNA*	pDR derivate consists of the promoter *P*_*HpaⅡ*_, *sgRNA*, *Tamy*, the up and downstream fragment of *prob* gene	This study
pHY-*ArgA*	pHY containing promoter *P*_*HpaⅡ*_, the gene *ArgA* and *T*_*amy*_ terminator	This study
pHY-*ArgB*	pHY containing promoter *P*_*HpaⅡ*_, the gene *ArgB* and *T*_*amy*_ terminator	This study
pHY-*ArgC*	pHY containing promoter *P*_*HpaⅡ*_, the gene *ArgC* and *T*_*amy*_ terminator	This study
pHY-*ArgD*	pHY containing promoter *P*_*HpaⅡ*_, the gene *ArgD* and *T*_*amy*_ terminator	This study
pHY-*ArgE*	pHY containing promoter *P*_*HpaⅡ*_, the gene *ArgE* and *T*_*amy*_ terminator	This study
pHY-*ArgA-ArgE*	pHY containing promoter *P*_*HpaⅡ*_, the gene *ArgA-ArgE* and *T*_*amy*_ terminator	This study
pHY-*ArgABCDE*	pHY containing promoter *P*_*HpaⅡ*_, the gene *ArgA, ArgB, ArgC, ArgD, ArgE* and *T*_*amy*_ terminator	This study
pHY- *Cg.lysE*	pHY containing promoter *P*_*HpaⅡ*_, the gene *Cg.lysE* and *T*_*amy*_ terminator	This study
pHY- *E.coli.lysE*	pHY containing promoter *P*_*HpaⅡ*_, the gene *E.coli.lysE* and *T*_*amy*_ terminator	This study
pHY- *BS.lysE*	pHY containing promoter *P*_*HpaⅡ*_, the gene *BS.lysE* and *T*_*amy*_ terminator	This study
pHY- *BA.lysE*	pHY containing promoter *P*_*HpaⅡ*_, the gene *BA.lysE* and *T*_*amy*_ terminator	This study
pHY-*ArgA*-*ArgE*- *BA.lysE*	pHY containing promoter *P*_*HpaⅡ*_, the gene *ArgA*-*ArgE*- *BA.lysE* and *T*_*amy*_ terminator	This study

### Media and Culture Conditions

For regular cloning and transformation experiments, *E. coli* and *B. amyloliquefaciens* strains were grown at 37°C in Luria-Bertani (LB) medium (10 g/L tryptone, 10 g/L yeast extract, and 5 g/L NaCl) containing the appropriate antibiotic. The composition of the initial fermentation medium was as follows: 80 g/L inulin, 40 g/L (NH_4_)_2_SO_4_, 20 g/L K_2_HPO_4_·3H_2_O, 2 g/L KH_2_PO_4_, 0.4 g/L MgSO_4_, 0.06 g/L MnSO_4_·H_2_O. The initial fermentation inoculum was 6%. For shake flask and batch fermentation, the cells were precultured in a 250 ml shake flask with 40 ml of seed culture and incubated at 37°C while shaking at 200 rpm for 12 h. For flask cultures, 2.5 ml of the seed culture was transferred into 500 ml flasks containing 50 ml of medium and cultured at 32°C and 200 rpm for 72 h. All fermentations in shake flasks were performed without the addition of exogenous sodium glutamate. During the fermentation process of L-ornithine, samples were collected periodically to evaluate the synthesis of L-ornithine and analyze bacterial growth by measuring optical density at 600 nm (OD_600_).

To obtain the optimal conditions for *B. amyloliquefaciens* to synthesize L-ornithine, we systematically optimized the fermentation conditions and medium composition in shake flasks. Some important factors (temperature, inoculum size, liquid volume, initial pH, carbon source, nitrogen source and metal ions) were selected to investigate their effects on L-ornithine synthesis. Firstly, 28°C, 30°C, 32°C, 34°C, 37°C and 40°C were selected to study the optimal temperature for L-ornithine fermentation. Secondly, we investigated the effect of the initial pH of the medium at 6.0, 7.0, 8.0, and 9.0 on L-ornithine synthesis, respectively. Then, the effects of inoculum size of 1%, 2%, 3%, 4%, 5%, and 6% on L-ornithine synthesis were investigated. Finally, the effects of adding 40 ml, 50 ml, 60 ml, 70 and 80 ml of fermentation broth to 500-ml shake flask on L-ornithine synthesis were investigated. In addition, the effects of the carbon source, nitrogen source and inorganic salt of the medium on l-ornithine synthesis were also investigated respectively. The Box–Behnken experimental design and response surface analysis were used to optimize the concentration of three key components in the L-ornithine fermentation medium: inulin, peptone, and MgSO_4_. The Statistica 7.0 software Experimental Design module was used to perform a quadratic polynomial regression fitting on the experimental data, and obtain the quadratic empirical equation model of L-ornithine on inulin, peptone, and MgSO_4_ (Y = 12.22 + 0.060A− 0.28B+ 0.17C + 0.087AB+ 0.027AC + 0.010BC − 1.64A^2^ − 1.29B^2^ − 1.35C^2^) (Y is the predicted value of L-ornithine production; A, B, and C are the coding values of inulin, peptone, and MgSO_4_, respectively) (Supplementary Tables S1–S3).

For batch fermentation in a 7.5 L fermenter, single colonies of engineered strains were picked and grown overnight at 32°C in LB medium, then inoculated at 1% (V/V) into 250 ml shake flasks containing 20 ml of seed medium for 12 h. Seed cultures were inoculated at 5% inoculum into a 7.5 L fermenter (BioFlo 115, New Brunswick Scientific, United States) with 4.5 L working volume. The stirring rate was set to 400 rpm, and the airflow was 1 vvm. To further improve the accumulation of L-ornithine, a fed-batch fermentation strategy was adopted. The feed solution with 20 g/L sodium glutamate were further fed into the fermenter at a constant flow rate during the fermentation period from 24 to 48 h.

### DNA Manipulation and Plasmid Construction

The primers used in this study are listed in Supplementary Table S4. The genome of *B. amyloliquefaciens* (NC_017190.1) was extracted using a bacterial total DNA extraction kit (Code DC103-01, Vazyme, Nanjing, China). DNA fragments of *argA* (Gene ID: 947289), *argB* (Gene ID: 56457340), *argC* (Gene ID: 56457338), *argD* (ID: 12201583), and *argE* (Gene ID: 948456) were obtained from this genome using primer pairs ArgA-F/R, ArgB-F/R, ArgC-F/R, ArgD-F/R and ArgE-F/R, respectively. The plasmid pHY (containing the constitutive strong promoter *P*_*HpaII*_, p15A ori, Cm^R^) was digested with restriction enzymes SalI/xhoI and purified by column (Code DC301-01, Vazyme, Nanjing, China). A*rgA, ArgB, ArgC, ArgD, ArgE, ArgABCDE* and *ArgA-ArgE* were ligated with the linearized pHY vector using ClonExpress II one-step cloning kit (Code C112-02-AB, Vazyme, Nanjing, China) to obtain recombinant plasmids pHY-ArgA, pHY-ArgB, pHY-ArgC, pHY-ArgD, pHY-ArgE, pHY-ArgABCDE and pHY-ArgA-ArgE, respectively. The genome of *B. amyloliquefaciens LL3*, *C. glutamicum*, *E. coli MG1655* and *B. subtilis 168* was extracted using a bacterial total DNA extraction kit (Code DC103-01, Vazyme, Nanjing, China). The genes encoding L-ornithine transporters *BA. lysE*, *Cg. lysE*, *E. coli.lysE* and *BS. lysE* were amplified from genomes of the different strains mentioned above with primer pairs BA. lysE-F/R, Cg. lysE-F/R, *E. coli*.lysE-F/R and BS. lysE-F/R, respectively. The recombinant plasmids pHY-BA. lysE, pHY-Cg. lysE, pHY-*E. coli*.lysE and pHY-BS. lysE were obtained by using the above-mentioned expression plasmid construction method. The constructed recombinant plasmids were verified by PCR and sanger sequencing.

The gene knockout method refers to previous research reports (Qiu et al., 2020b). To knock out *argF*, upstream and downstream fragments of *argF* (Gene ID: 56457344) were amplified from *B. amyloliquefaciens* genome (NC_017190.1) with primer pairs argFL-F/R and argFR-F/R, respectively. sgRNA of *argF* was designed using online software (http://cistrome.org/SSC/) and obtained by designing primers sgargF-F/R. The DNA fragments amplified above were ligated by overlapping PCR to obtain argFsgRNA-argFL-argFR. The fragment argFsgRNA-argFL-argFR was then ligated with the linearized pDR-*uppsgRNA* vector (linearized with restriction enzymes salI/xhoI) using ClonExpress II one-step cloning kit (Code C112-02-AB, Vazyme, Nanjing, China) to obtain recombinant plasmids pDR-*argFsgRNA*. The *argI* (Gene ID: 12203901), *speF* (Gene ID: 945297) and *prob* (Gene ID: 56457565) genes of *B. amyloliquefaciens* were knocked out using the above method to construct different engineered strains. The constructed recombinant plasmids were verified by colony PCR and sanger sequencing.

### Transformation Method of *B. amyloliquefaciens*

The transformation of wild-type *B. amyloliquefaciens* to *B. amyloliquefaciens* NB was performed using a modified high-osmolarity electroporation method (Sha et al., 2019)**.** An overnight culture of *B. amyloliquefaciens* was diluted 100-fold in fresh medium (LB broth containing 0.5 M sorbitol) to prepare electrocompetent cells. When the OD_600_ of the culture reached 0.5, the NB cells were harvested by centrifugation at 4°C and 8,000 rpm for 10 min. After four washes in ice-cold electroporation medium (0.5 M sorbitol, 0.5 M mannitol, and 10% glycerol), the electrocompetent cells were suspended at a cell density of 1 × 10^10^ colony-forming units/mL.

### Analysis of Cell Growth, Glutamate Levels, ATP Levels, and L-Ornithine Content

The optical density of a sample was measured to determine cell growth. The OD_600_ of the diluted sample was measured using a spectrophotometer. The glutamate level was measured using Glutamate Content Assay Kit (Code MS 1906, Shanghai, China) from Shanghai Suoqiao Biological Co., Ltd. The detection principle of the kit is that the enzyme reagent can specifically recognize glutamate substrate from a mixture and catalyze it to produce a colored product, and the reaction product has a maximum absorption peak at a wavelength of 570 nm. The ATP content was determined using the ATP content kit (phosphomolybdic acid colorimetry) (Code G0815W, Suzhou, China) purchased from Suzhou Grace Biotechnology Co., Ltd. The creatine kinase in the kit can catalyze the ATP reaction with creatine to produce creatine phosphate, which is detected by the phosphomolybdic acid colorimetric method. Therefore, the ATP content can be calculated based on the maximum absorption peak of the reaction product at 700 nm. The L-ornithine content was detected through Chinard’s L-ornithine measurement method. Specifically, 6 mol/L H_3_PO_4_-glacial acetic acid (1/3, v/v) was used to prepare a 25 mg/ml ninhydrin solution as the coloring solution. After the coloring solution reacted with L-ornithine in a water bath at 100°C for 60 min, the absorption peak was measured at 510 nm (Shu et al., 2018a).

## Results

### Feasibility Analysis of L-Ornithine Production by *B. amyloliquefaciens* NB

**FIGURE 1:**
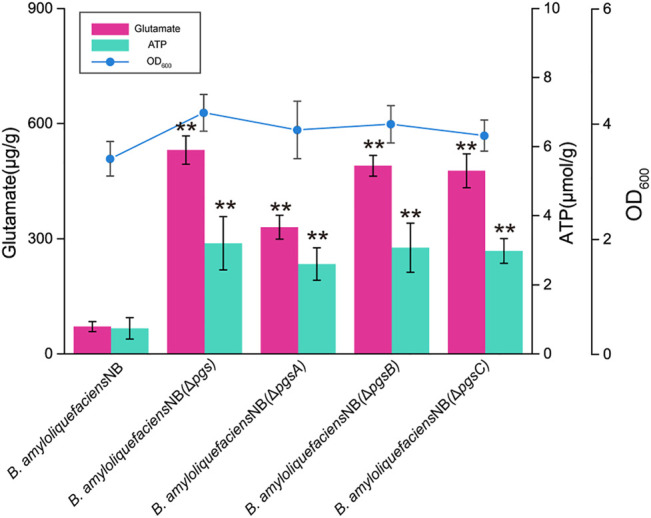
The lack of polyglutamate synthase (*pgsBCA*) improves glutamate and ATP concentrations and growth. Biomass and glutamate and ATP concentrations for *B. amyloliquefaciens* NB, *B. amyloliquefaciens* (*ΔpgsBCA*), *B. amyloliquefaciens* (*ΔpgsA*), *B. amyloliquefaciens* (*ΔpgsB*), and *B. amyloliquefaciens* (*ΔpgsC*) in shake flasks at 24 h*.* All data were the average of three independent studies with standard deviations. The ** and * indicate *p* < 0.01 and 0.05 relative to the control strain *B. amyloliquefaciens* NB, respectively.

In an earlier study, we found that *B. amyloliquefaciens* NB can efficiently use inulin to synthesize PGA and has a highly-efficient tricarboxylic acid cycle metabolic flux and glutamate synthesis ability (Qiu et al., 2020a; Sha et al., 2020b). Therefore, we speculate that this strain may be optimal for synthesizing glutamate derivatives (L-ornithine). First, the polyglutamate synthase *pgsBCA* of *B. amyloliquefaciens* NB was knocked out to prevent the synthesis of γ-PGA from glutamate and release ATP for the synthesis of γ-PGA. After *pgsBCA* was knocked out, the colony morphology of *B. amyloliquefaciens* NB changed from wet to rough and almost no γ-PGA was detected in the fermentation broth of *B. amyloliquefaciens* NB (Δ*pgsBCA*), but 0.43 g/L of L-ornithine was obtained (Almost no L-ornithine was present in the fermentation broth of the original strain) (Supplementary Figures S1, S2). In addition, compared with *B. amyloliquefaciens* NB, the intracellular glutamate and ATP levels of *B. amyloliquefaciens* NB (Δ*pgsBCA*) increased by 7 times and 4.5 times, respectively (Figure 1). By blocking the synthesis of γ-PGA, the intracellular glutamate and ATP content of *B. amyloliquefaciens* NB (Δ*pgsBCA*) were increased to 531 μg/g and 3.2 μmol/g, respectively. In addition, the growth of *B. amyloliquefaciens* NB was also improved, and its OD_600_ increased from 3.2 to 4.3. These results demonstrated that *B. amyloliquefaciens* NB has certain advantages as an L-ornithine-producing strain. However, glutamate represents a critical node in many important metabolic pathways, and it is an essential intermediate of many products. For example, glutamate is a precursor of proline, L-ornithine, and arginine (Jiang et al., 2021; Tran et al., 2021). Therefore, achieving efficient glutamate conversion to L-ornithin in *B. amyloliquefaciens* is a fundamental challenge for obtaining a high-efficiency strain.

### Modular Optimization of Metabolic Pathways to Enhance L-Ornithine Synthesis

**FIGURE 2:**
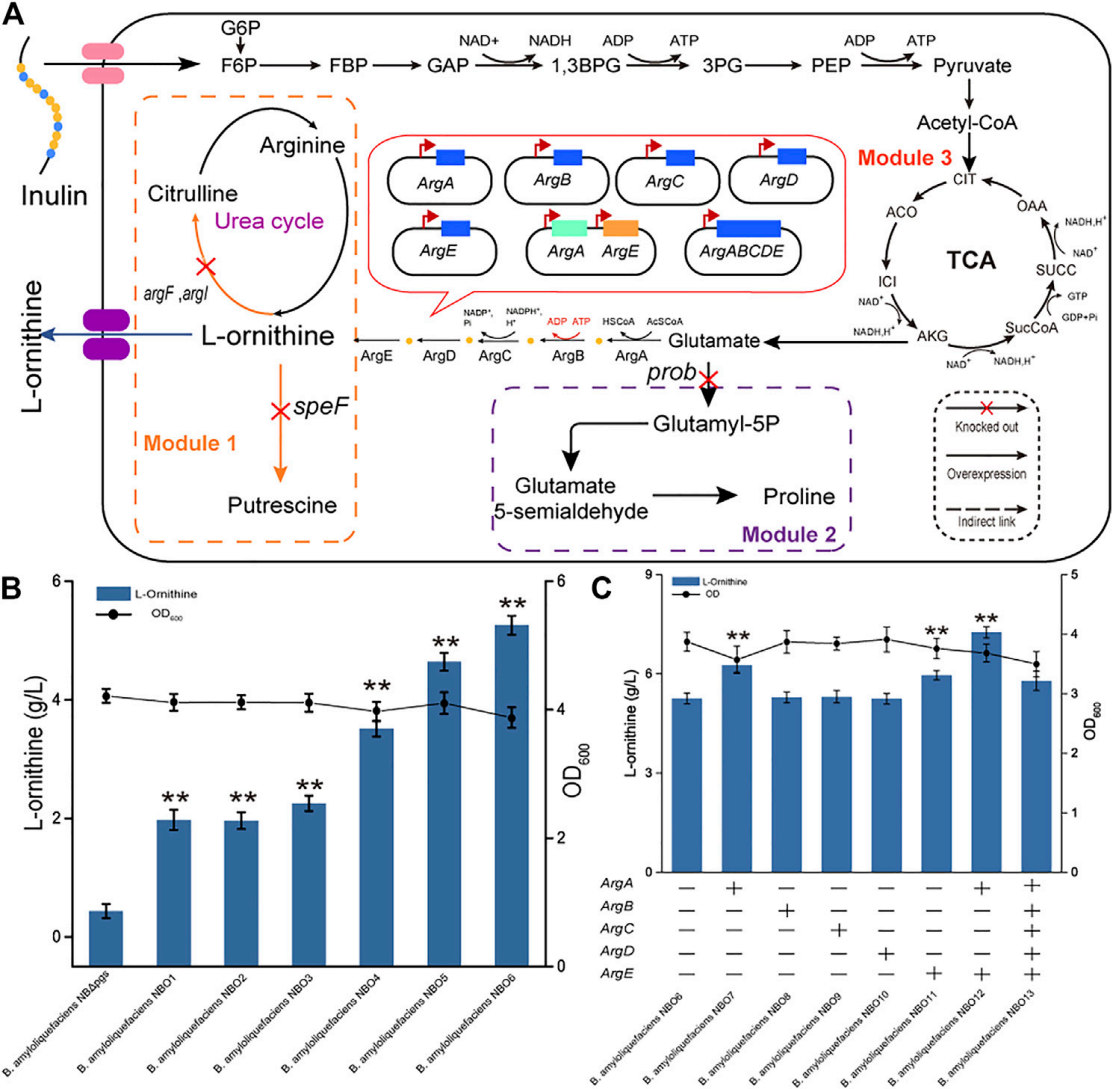
Effect of the L-ornithine synthesis module on putrescine production. **(A)** Schematic overview of L-ornithine catabolism pathway, precursor competition pathway, and synthesis pathways. The main pathway of l-ornithine catabolism is catalyzed by L-ornithine carbamoyltransferase and l-ornithine decarboxylase. Glutamate is a key starting material for L-ornithine synthesis. Blocking proline synthesis improves the supply of the precursor glutamate for L-ornithine synthesis. Some key genes in the L-ornithine synthesis pathway included amino-acid N-acetyltransferase (ArgA), acetylglutamate kinase (ArgB), N-acetyl-gamma-glutamyl-phosphate reductase (ArgC), acetylornithine aminotransferase (ArgD), and acetylornithine deacetylase (ArgE). G6P: Glucose-6-Phosphate; F6P: Frucose-6- Phosphate; GAP: Glyceraldedyde-3-phosphate; 1,3BPG: 1,3-Bisphospho-glyerate; 3 PG: 3-phosphoglycerate; PEP: Phosphoenolpyruvate **(B)** The L-ornithine titers of the recombinant strains lacking *argF*, *argI*, *speF*, and *prob*. All data were the average of three independent studies with standard deviations. The ** and * indicate *p* < 0.01 and 0.05 relative to the control strain *B. amyloliquefaciens* NB (*ΔpgsBCA*), respectively. **(C)** Overexpression of the L-ornithine synthase gene in *B. amyloliquefaciens* NBO6. “+” indicates that the relevant gene is overexpressed in the strain; “-” indicates that the relevant gene is not overexpressed in the strain. All data were the average of three independent studies with standard deviations. The ** and * indicate *p* < 0.01 and 0.05 relative to the control strain *B. amyloliquefaciens* NBO6, respectively. Co-expression of genes with noticeable effect to further improve the fermentation effect.

Optimizing the L-ornithine synthesis pathway involves multiple metabolic pathways: the L-ornithine degradation pathway, the precursor competition pathway, and the L-ornithine synthesis pathway. These need to be coordinated to prompt the synthesis L-ornithine from glutamate. We divided these pathways into three modules, namely, module one (L-ornithine catabolism), module two (precursor competition), and module three (L-ornithine synthesis) (Figure 2A).

As a precursor, L-ornithine could be converted into citrulline, putrescine, and other substances in the cell. For module 1, the *argF* and *argI* genes encoding L-ornithine carbamoyltransferase (Sander et al., 2019). were first knocked out to block the catabolism of L-ornithine to citrulline, thereby obtaining strains *B. amyloliquefaciens* NBO1 and NBO2. The fermentation results showed that blocking these two genes promoted the accumulation of L-ornithine; the L-ornithine titers of the NBO1 and NBO2 strains reached 1.97 g/L and 1.95 g/L, respectively. In addition, the *speF* gene, encoding L-ornithine decarboxylase (Bao et al., 2021), was knocked out to block the catabolism of L-ornithine to putrescine in *B. amyloliquefaciens* NBO3 [with *argF* and *argI* genes deleted in *B. amyloliquefaciens NB* (Δ*pgsBCA*)], thereby obtaining the *B. amyloliquefaciens* strain NBO4. The L-ornithine titer of NBO4 was further increased to 3.51 g/L (Figure 2B).

Glutamate is essential for L-ornithine synthesis and is a key starting material for other metabolic pathways (Xu et al., 2019). Therefore, the metabolic flow of glutamate directly affects the synthesis efficiency of L-ornithine. For module 2, the *prob-*encoded glutamate 5-kinase was knocked out to block proline synthesis, which competes with L-ornithine synthesis for the precursor glutamate. *B. amyloliquefaciens* NBO5 *and* NBO6 were obtained by knocking out the *prob* gene (ID: 56457565) in *B. amyloliquefaciens* NBO3 and NBO4, respectively. The results showed that the deletion of glutamate 5-kinase produced the most significant increase in the titer of L-ornithine. The titer of L-ornithine in *B. amyloliquefaciens* NBO5 and NBO6 reached 4.64 and 5.26 g/L, respectively (Figure 2B).

Previous studies have reported that the L-ornithine synthase cluster is an important factor limiting the efficient synthesis of L-ornithine. For example, the rate-limiting steps in the L-ornithine synthesis pathways of *C. glutamicum* and *E. coli* are N-acetylglutamate synthase encoded by *argA* and N-acetylglutamate kinase encoded by *argB*, respectively (Yoshida et al., 1979; Rajagopal et al., 1998). Higher yields can be obtained by overexpressing related genes using high-copy plasmid vectors, etc. Therefore, it is imperative to study the expression of L-ornithine synthase for efficient L-ornithine synthesis. For module 3, we studied the effects of the following enzymes on L-ornithine synthesis: amino-acid N-acetyltransferase (*ArgA*), acetylglutamate kinase (*ArgB*), N-acetyl-gamma-glutamyl-phosphate reductase (*ArgC*), acetylornithine aminotransferase (*ArgD*), and acetylornithine deacetylase (*ArgE*). These enzymes were expressed in strain *B. amyloliquefaciens* NBO6, respectively, resulting in engineering strains *B. amyloliquefaciens* NBO7, NBO8, NBO9, NBO10, and NBO11. The fermentation results of five recombinant strains and the control strain (*B. amyloliquefaciens* NBO6) showed that the overexpression of *ArgA* and *ArgE* contributed to L-ornithine synthesis. Subsequently, we co-overexpressed *ArgA* and *ArgE* in *B. amyloliquefaciens* NBO6 and found that the titer of L-ornithine of NBO12 was further improved, reaching 7.26 g/L. However, co-overexpression of *ArgA, ArgB, ArgC, ArgD* and *ArgE* in *B. amyloliquefaciens* NBO6 did not significantly improve L-ornithine production, and the production of *B. amyloliquefaciens* NBO13 was lower than that of NBO12. Overall, by optimizing the three modules, the production of L-ornithine synthesized by *B. amyloliquefaciens* using inulin as a substrate was increased almost 17-fold from 0.43 to 7.26 g/L (Figure 2C).

### Screening the L-Ornithine Transporter to Enhance the Extracellular Accumulation of L-Ornithine

**FIGURE 3:**
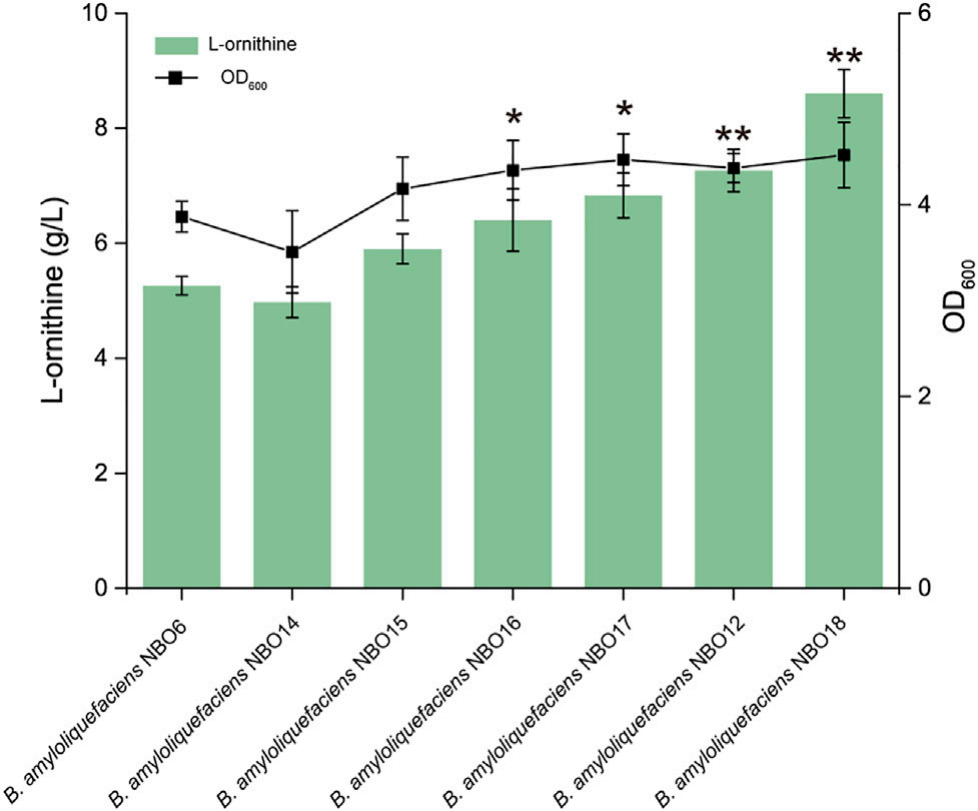
The expression of *lysE* increases L-ornithine synthesis and cell growth. The effect of the overexpression of four transporter genes on L-ornithine production and cell growth. Four L-ornithine transporters from different sources were screened, namely, *E. coli*, *C. glutamicum*, *B. subtilis*, and *B. amyloliquefaciens*. All data were the average of three independent studies with standard deviations. The ** and * indicate *p* < 0.01 and 0.05 relative to the control strain *B. amyloliquefaciens* NBO6, respectively.

Products accumulated in the cell cause feedback inhibition on the activity of critical enzymes, which can have detrimental effects on cell growth (Luo et al., 2018). Therefore, accelerating the extracellular transport efficiency of l-ornithine is very important to enhance the extracellular accumulation of L-ornithine and alleviate its feedback inhibition on the synthesis pathway. First, the gene *lysE* (encoding the L-ornithine transporter) from different strains (*E. coli*, *C. glutamicum*, *B. subtilis*, and *B. amyloliquefaciens*) was overexpressed in NBO6 to screen high-efficiency transporters. The results indicated that L-ornithine production of the strains expressing the transporter was improved at different levels compared to the control strains (Figure 3). Among them, the overexpression of *lysE* from *B. amyloliquefaciens* (*BA.lysE*) resulted in a significant increase in L-ornithine titer and biomass. The titer of L-ornithine increased by 30% compared with NBO6, reaching 6.83 g/L. Subsequently, the *lysE* from *B. amyloliquefaciens* was further overexpressed in *B. amyloliquefaciens* NBO12 to enhance L-ornithine production, resulting in *B. amyloliquefaciens* NBO18. The L-ornithine titer of *B. amyloliquefaciens* NBO18 reached 8.6 g/L, representing a 20% increase compared with *B. amyloliquefaciens* NBO12 (Figure 3).

### Optimization of the Fermentation Process to Further Improve L-Ornithine Synthesis

**FIGURE 4:**
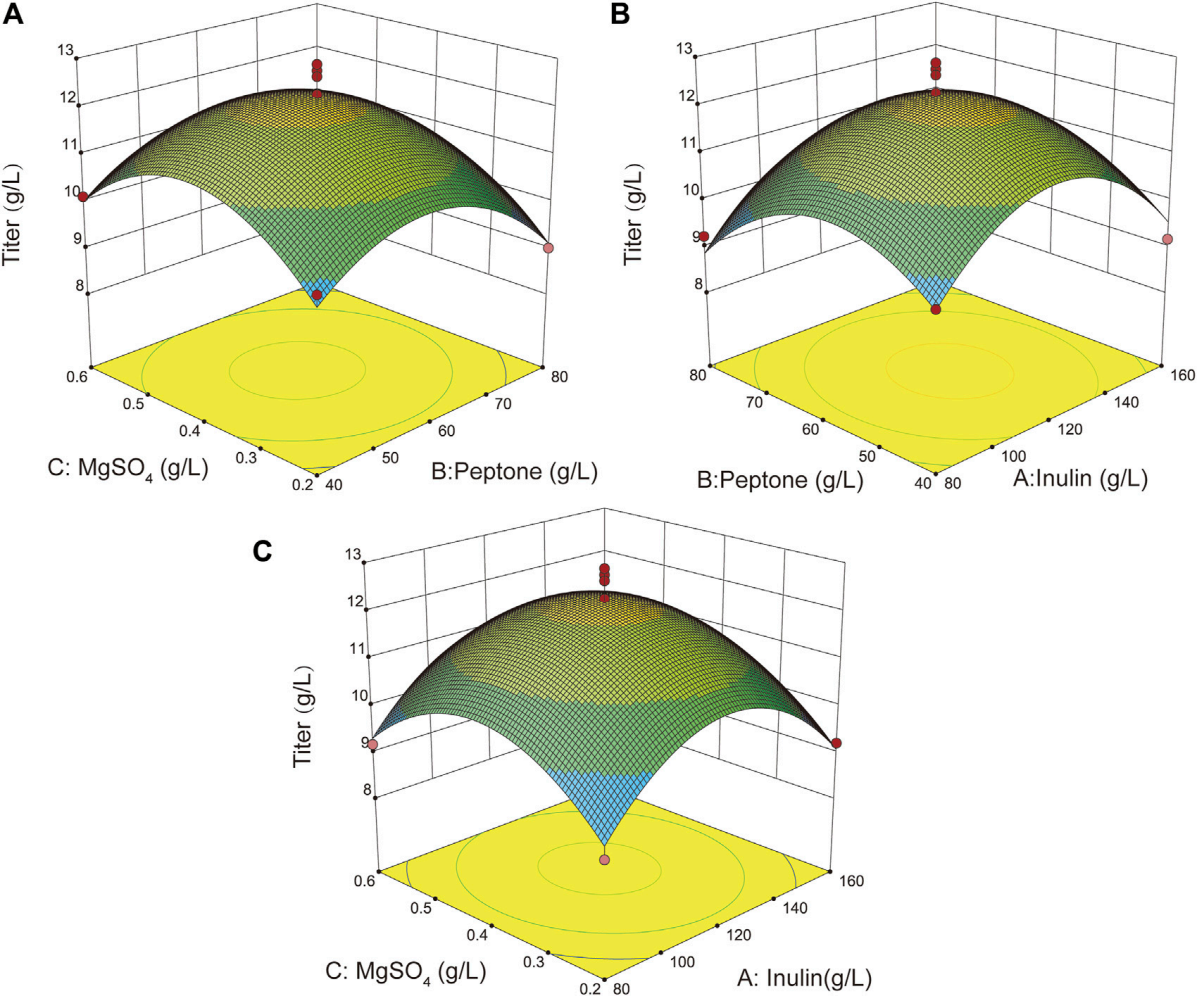
The effect of different fermentation conditions on L-ornithine production and response surface optimization results. Three-factor and three-level response surface optimization for three major components (inulin, peptone, and MgSO_4_) was done. **(A)** The response surface plot showed the effects of MgSO_4_ and peptone on l-ornithine production. **(B)** The response surface plot showed the effects of inulin and peptone on L-ornithine production. **(C)** The response surface plot showed the effects of inulin and MgSO_4_ on L-ornithine production.

The fermentation process and medium components are critical for strain growth and for the synthesis of the desired products (Luo et al., 2020). Therefore, it is essential to optimize the fermentation process and medium composition to further enhance the accumulation of L-ornithine. First, the inoculum volume, liquid volume, temperature, and pH of the *B. amyloliquefaciens* NBO18 strain were optimized. The fermentation results showed that the inoculum volume, liquid volume, temperature, and pH were 5%, 10%, 32°C, and 7.0, respectively (Supplementary Figure S3). In addition, the composition of the fermentation medium was further optimized by combining single factor and response surface experiments (Supplementary Figure S3), and the best carbon source, nitrogen source, and metal ions were determined, which were 120 g/L of inulin, 60 g/L of peptone, and 0.4 g/L of MgSO_4_, respectively. The titer of L-ornithine of *B. amyloliquefaciens* NBO18 under optimal fermentation conditions reached 12.6 g/L, representing a 31.7% increase (Figure 4).

### Maximizing L-Ornithine Production in a 7.5 L Fermenter

**FIGURE 5:**
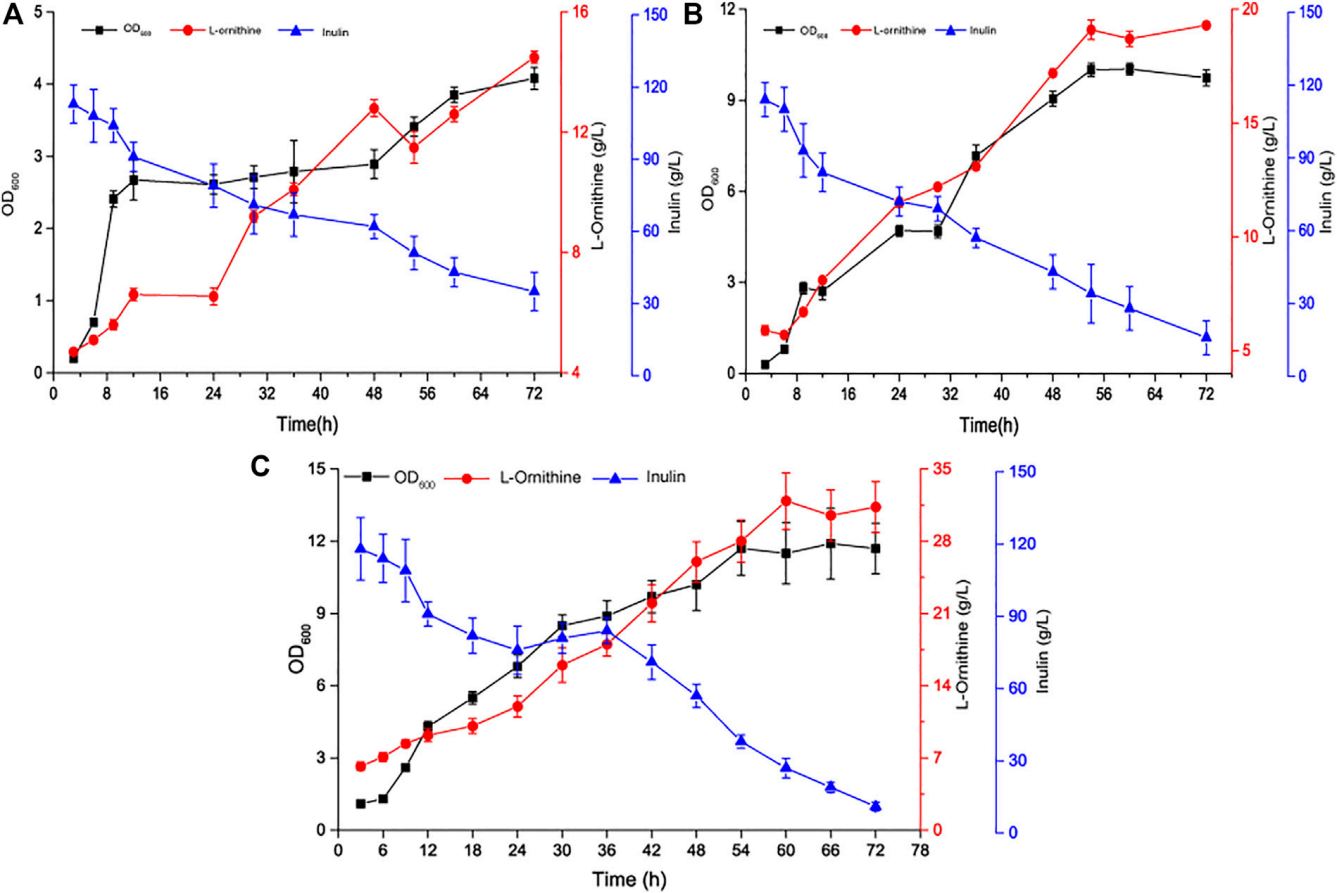
The time curve of L-ornithine fermentation in a 7.5 L fermenter. **(A)** Time profile of single-batch L-ornithine fermentation using inulin as the carbon source in a 7.5 L fermenter. **(B)** Time profile of single-batch L-ornithine fermentation using inulin and sodium glutamate as the carbon source in a 7.5 L fermenter. **(C)** Time profile of fed-batch L-ornithine fermentation in a 7.5 L fermenter. Fermentation conditions are as follows: the working volume is 3.5-L, the stirring rate is 400 rpm, the volume air per volume is 1 vvm, and the inoculation size is 6%.

To maximize L-ornithine production by *B. amyloliquefaciens* NBO18, we cultured NBO18 in 7.5 L batch bioreactors (BioFlo 115, New Brunswick Scientific, United States) based on the optimized fermentation conditions above, resulting in titers of 14.5 g/L L-ornithine (Figure 5A). To further improve L-ornithine production, 20 g/L of the precursor sodium glutamate was added to L-ornithine batch fermentation, and 19.3 g/L of L-ornithine was obtained (Figure 5B). However, the efficiency of sodium glutamate conversion into L-ornithine was very low, which may explain why the high concentration of sodium glutamate had a negative effect on strain metabolism. Thus, the sodium glutamate feeding strategy was adopted, and sodium glutamate was added to the fermenter at a constant flow rate of 0.5 ml/min during a fermentation period of 24–48 h (the final supplement amount of sodium glutamate was about 20 g/L). Finally, the titer of L-ornithine produced by *B. amyloliquefaciens* NBO18 reached 31.3 g/L, and the yield of L-ornithine was 0.22 g/g (L-ornithine/(inulin + glutamate)) (Figure 5C).

## Discussion

Using non-grain raw materials to synthesize biochemical products is very difficult due to the inefficiency of their bio-utilization. Although much work has been done to solve this issue, the current efficiency of synthesizing biochemical products from non-grain raw materials is lower than that of synthesizing biochemical products from food raw material substrates such as glucose (Qiu et al., 2019a; Becker and Wittmann, 2019). Here, we systematically studied the biosynthesis of L-ornithine by *B. amyloliquefaciens* using non-grain food materials. First, we demonstrated the feasibility of fermenting L-ornithine from Jerusalem artichoke by analyzing intracellular glutamate and ATP levels. Then, modular engineering and carrier engineering were applied to prompt high levels of intracellular precursor glutamate conversion into L-ornithine. This enabled *B. amyloliquefaciens* to efficiently produce L-ornithine from Jerusalem artichoke without glutamate supplementation. Combining these strategies with an optimized fermentation process, we successfully achieved a final titer of 31.3 g/L L-ornithine. We anticipate that these strategies should be widely applicable in the microbial synthesis of value-added glutamate derivatives by *B. amyloliquefaciens* using non-grain food materials.

To our knowledge, this is the first report of the use of non-grain food materials to produce L-ornithine using *B. amyloliquefaciens*. In previous studies, a small amount of L-ornithine was synthesized by model strains (*E. coli*, *C. glutamicum*) via fermentation of food raw materials (glucose and starch) (Lee and Cho, 2006; Wu et al., 2020). Compared with these model strains, *B. amyloliquefaciens* NB is advantageous since it serves as a cell factory for L-ornithine synthesis. We found that the intracellular glutamate content of *B. amyloliquefaciens* was significantly increased several times by blocking the PGA synthesis pathway. This demonstrates that the strain has an efficient glutamate synthesis flux and provides a sufficient precursor supply for L-ornithine synthesis. In addition, we compared the effects of different carbon sources on L-ornithine synthesis and found that inulin as a non-grain raw material was the dominant carbon source for L-ornithine synthesis compared with glucose, fructose, and other carbon sources. Overall, these results demonstrated that *B. amyloliquefaciens* could be optimal for the synthesis of glutamate derivatives from non-grain inulin. However, the synthesis of L-ornithine from inulin in this study requires a large amount of peptone and glutamate supplementation, which leads to an increase in the cost of producing L-ornithine. Therefore, it is necessary to rationally regulate the nitrogen metabolism pathway of *B. amyloliquefaciens* to improve its utilization efficiency of cheap nitrogen sources in the future.

Coordinated optimization of multiple pathways is essential for constructing efficient cell factories (Gong et al., 2020; Zhou et al., 2021). Most studies on the construction of L-ornithine cell factories thus far have focused on enhancing L-ornithine synthesis pathways but rarely on regulating the overall L-ornithine synthesis pathway (Shu et al., 2018b; Zhang et al., 2018). Although implementing these strategies improved L-ornithine production, local regulation of the metabolic pathway will cause an imbalance of the metabolic network limiting target product production (Wu et al., 2020). In this study, we systematically investigated and coordinated the optimization of the L-ornithine degradation pathway, precursor competing pathway, L-ornithine synthesis pathway and L-ornithine transport pathway. This enabled the production of a strain that efficiently utilized inulin to synthesize L-ornithine. In addition, the L-ornithine fermentation process was systematically optimized to further improve L-ornithine synthesis efficiency. Finally, the titer of L-ornithine increased from 0.43 to 31.3 g/L. These results demonstrate that systematically optimizing the metabolic network of strains is invaluable for efficient synthesis of target products. In addition, the strategies employed in this study could prove useful for constructing high-efficiency cell factories of glutamate and its related products.

Efficient utilization of non-grain raw materials is a crucial challenge, hampering efficient synthesis of target products by microbial strains (Kamimura et al., 2019). Surprisingly, we found that the utilization efficiency of inulin by microorganisms was significantly higher than that of other non-grain materials such as cellulose, hemicellulose, and lignin (Shu et al., 2018b; Cai et al., 2021). This may be because *B. amyloliquefaciens* NB has a highly active inulin degrading enzyme, enabling efficient inulin degradation into fermentable monosaccharides (glucose and fructose) (Qiu et al., 2019a). Therefore, the inulin utilization module pathway from *B. amyloliquefaciens* NB could be designed in model microbial cells to achieve efficient synthesis of target products using inulin non-food raw materials. Nonetheless, the conversion rate of L-ornithine synthesized by *B. amyloliquefaciens* from non-grain raw materials was lower than that of a model strain using food grain as the raw material and a theoretical conversion rate (Sheng et al., 2021). The main reason may be that the mechanism for efficient inulin utilization remains unclear. For example, key factors affecting strain metabolism and growth remain unclear. Furthermore, the mechanism for coordinated utilization of fructose and glucose from inulin has not been elucidated. Therefore, the effects of these factors on strain metabolism should be further analyzed. Moreover, the conversion rate of inulin into target products requires improvement in future studies, since this will play a vital role in the utilization efficiency of other complex carbon sources.

## Data Availability

The original contributions presented in the study are included in the article/Supplementary Material, further inquiries can be directed to the corresponding author.
