# Supplementary material for: Metabolic Engineering of Bacillus amyloliquefaciens to Efficiently Synthesize L-Ornithine From Inulin
Source: Front Bioeng Biotechnol. 2022 Jun 8;10:905110. doi: 10.3389/fbioe.2022.905110 (PMC9214239; doi:10.3389/fbioe.2022.905110)
Supplement: Supplementary file 1 [file DataSheet1.docx]

**Supplementary material**

**Metabolic engineering of *Bacillus amyloliquefaciens* to efficiently synthesize l-ornithine from inulin**

Yifan Zhu^†,‡^, Yi Hu^†,‡^, Yifan Yan^†,‡^, Shanshan Du^†,‡^, Fei Pan^†,‡^, Sha Li^†,‡^, Hong Xu^†,‡^, Zhengshan Luo^†,‡*^

^†^ State Key Laboratory of Materials-Oriented Chemical Engineering, Nanjing Tech University, Nanjing 211816, China

^‡^ College of Food Science and Light Industry, Nanjing Tech University, Nanjing 211816, China

* **Corresponding author.**

Zhengshan Luo

Mailing address: College of Food Science and Light Industry, Nanjing Tech University, 30 Puzhu Road, Nanjing, Jiangsu 211816, China

Phone: +86-025-58139433, Fax: +86-025-58139433

E-mail: [luozs@njtech.edu.cn](mailto:luozs@njtech.edu.cn)

ORCID iD: <https://orcid.org/0000-0002-3087-5663>

# Supplementary Figures


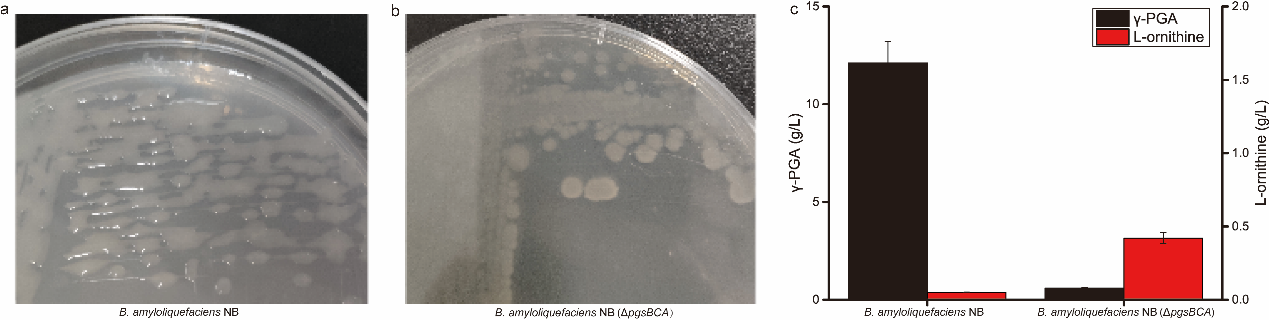


## Figure. S1 Comparison of colony morphology and fermentation products of strains before and after knockout of polyglutamate synthase gene *pgsBCA*.

(a) Colony morphology of *B. amyloliquefaciens* NB (control strain, without *pgsBCA* knockout) on LB solid plates. (b) Colony morphology of *B. amyloliquefaciens NB (ΔpgsBCA)* on LB solid plates. (c) Comparison of fermentation broth products of *B. amyloliquefaciens* NB and *B. amyloliquefaciens NB (ΔpgsBCA)*.


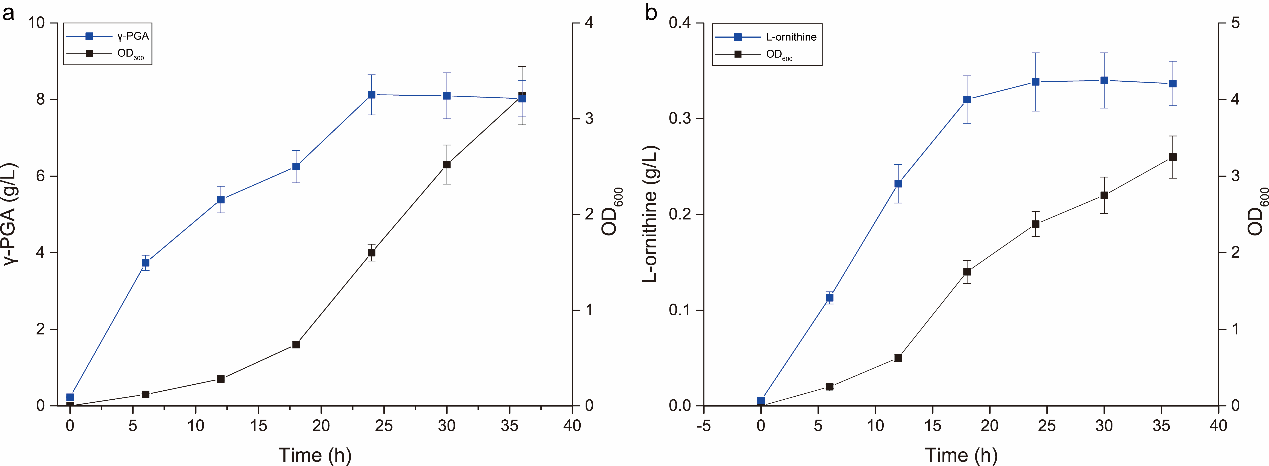


## Figure.S2 the growth and product synthesis curve of *B. amyloliquefaciens* NB and *B. amyloliquefaciens* NB (*ΔpgsBCA*).

(a) the growth and product synthesis curve of *B. amyloliquefaciens* NB. (b) the growth and product synthesis curve of *B. amyloliquefaciens* NB (*ΔpgsBCA*).


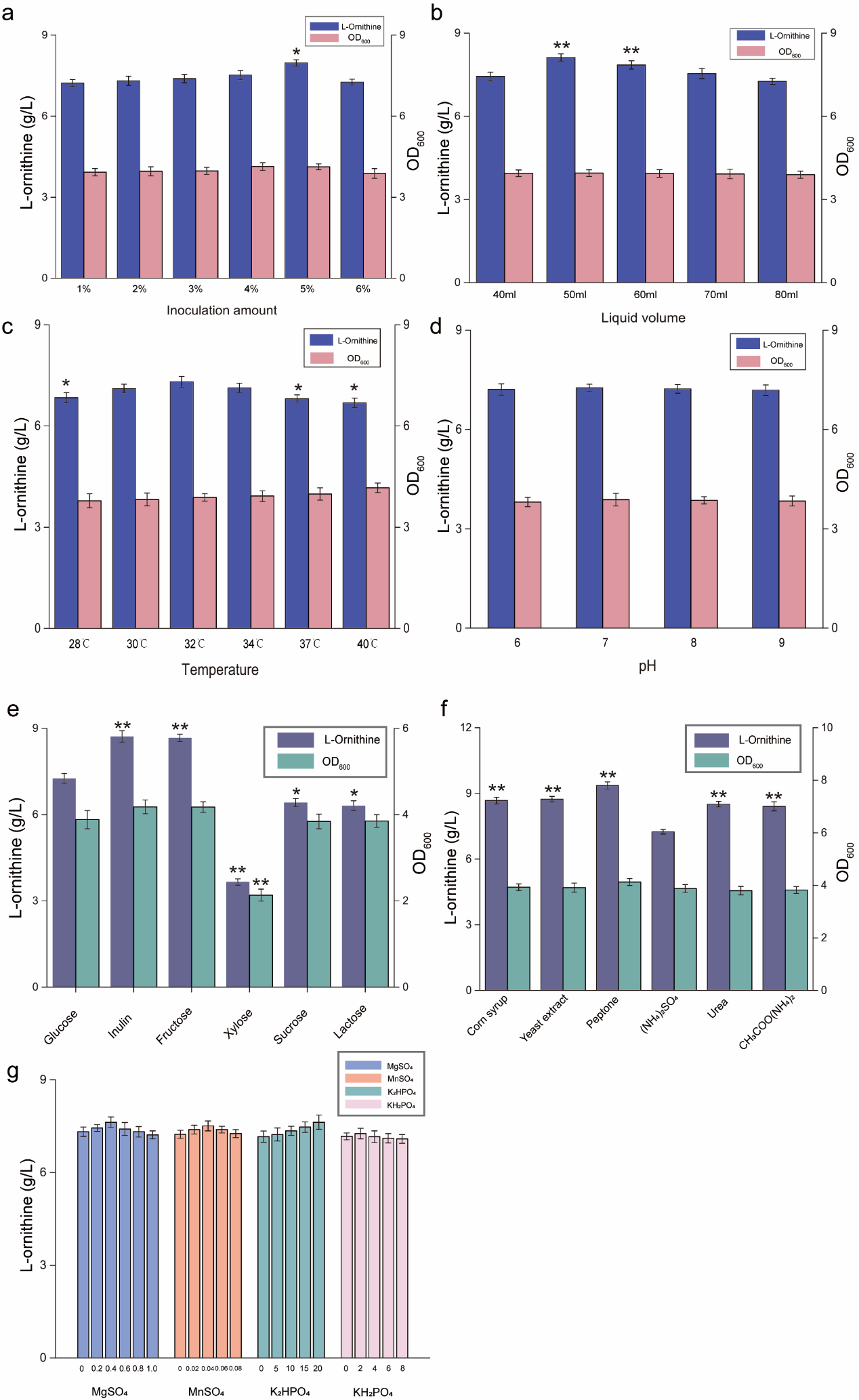


## Figure. S3 Effects of optimization of fermentation conditions and medium composition on l-ornithine synthesis

(a) Effect of inoculation amount on l-ornithine production. All data were the average of three independent studies with standard deviations. The ** and * indicate p<0.01 and 0.05 relative to the control condition 6% inoculation amount, respectively. (b) Effect of liquid volume on l-ornithine production. All data were the average of three independent studies with standard deviations. The ** and * indicate p<0.01 and 0.05 relative to the control condition 80 ml, respectively. (c) Effect of temperature on l-ornithine production. All data were the average of three independent studies with standard deviations. The ** and * indicate p<0.01 and 0.05 relative to the control condition 32℃, respectively. (d) Effect of pH on l-ornithine production. All data were the average of three independent studies with standard deviations. The ** and * indicate p<0.01 and 0.05 relative to the control condition pH 7.0, respectively. (e) Effect of carbon source on l-ornithine production. All data were the average of three independent studies with standard deviations. The ** and * indicate p<0.01 and 0.05 relative to the control carbon source (glucose), respectively. (f) Effect of nitrogen source on l-ornithine production. All data were the average of three independent studies with standard deviations. The ** and * indicate p<0.01 and 0.05 relative to the control nitrogen source ((NH_4_)_2_SO_4_), respectively. (g) Effect of metal ions on l-ornithine production. All data were the average of three independent studies with standard deviations. The ** and * indicate p<0.01 and 0.05 relative to the control condition, respectively.

# Supplementary Tables

## Table S1. Values of different factors.

| **Level*** | **Inulin (A)** | **Peptone (B)** | **MgSO_4_(C)** |
| --- | --- | --- | --- |
| -1 | 80 | 40 | 0.2 |
| 0 | 120 | 60 | 0.4 |
| 1 | 160 | 80 | 0.6 |

* Level refers to the different additions of each factor. -1, 0, 1 represent low, medium, and high, respectively.

## Table S2. The number of replicas performed.

| **Run** | **Inulin (g/L)** | **Peptone (g/L)** | **MgSO_4_ (g/L)** |
| --- | --- | --- | --- |
| 17 | 80 | 80 | 0.4 |
| 16 | 80 | 40 | 0.4 |
| 15 | 120 | 60 | 0.4 |
| 14 | 160 | 60 | 0.6 |
| 13 | 120 | 60 | 0.4 |
| 12 | 120 | 80 | 0.6 |
| 11 | 80 | 60 | 0.2 |
| 10 | 120 | 60 | 0.4 |
| 9 | 120 | 40 | 0.6 |
| 8 | 120 | 60 | 0.4 |
| 7 | 160 | 80 | 0.4 |
| 6 | 160 | 40 | 0.4 |
| 5 | 120 | 40 | 0.2 |
| 4 | 120 | 60 | 0.4 |
| 3 | 80 | 60 | 0.6 |
| 2 | 120 | 80 | 0.2 |
| 1 | 160 | 60 | 0.2 |

## Table S3. ANOVA for Response Surface Quadratic model.

| **Source** | **Sum of squares** | **df** | **Mean squares** | **F Value** | **P Value** |
| --- | --- | --- | --- | --- | --- |
| Model | 30.04 | 9 | 3.34 | 5.61 | 0.016 |
| A | 0.03 | 1 | 0.03 | 0.05 | 0.83 |
| B | 0.62 | 1 | 0.62 | 1.05 | 0.34 |
| C | 0.24 | 1 | 0.24 | 0.41 | 0.54 |
| AB | 0.03 | 1 | 0.03 | 0.05 | 0.83 |
| AC | 0.003 | 1 | 0.003 | 0.01 | 0.95 |
| BC | 0.0004 | 1 | 0.0004 | 0.0007 | 0.98 |
| A^2^ | 11.35 | 1 | 11.35 | 19.11 | 0.003 |
| B^2^ | 7.01 | 1 | 7.01 | 11.79 | 0.01 |
| C^2^ | 7.73 | 1 | 7.73 | 13.01 | 0.009 |
| Residual | 4.16 | 7 | 0.59 | - | - |
| Lack of Fit | 0.58 | 3 | 0.19 | 0.22 | 0.88 |
| Pure Error | 3.58 | 4 | 0.89 | - | - |
| Cor Total | 34.20 | 16 |  | - | - |

## Table S4. Primers and their sequences used for PCR in this Study.

| **Primers** | **Sguences(5’-3’)** |
| --- | --- |
| sgpgsB-F | AAAAAGGAGCGATTTAGTCGACAAGAAAAAGATGCAATCATAGTTTTAGAGCTAGAAAT |
| sgpgsB-R | GCTGAGATTTTTCGCCTTTATGATAAAAAAGCACCGACTCGGTGCCAC |
| pgsBL-F | GTGGCACCGAGTCGGTGCTTTTTTCATAAAGGCGAAAAATCTCAGC |
| pgsBL-R | CGTAAACGGCTTTGCCGCAAATGAATGCAGGAACTGATGTGTGTTTGC |
| pgsBR-F | GCAAACACACATCAGTTCCTGCATTCATTTGCGGCAAAGCCGTTTACG |
| pgsBR-R | AGGTGTTTTTTTATTACCCTCGAG ATGTGGTTACTCATTATAGCCTGT |
| sgpgsC-F | AAAAAGGAGCGATTTAGTCGACTCCGTACAAAATCATAAATCGTTTTAGAGCTAGAAATAGCAAGT |
| sgpgsC-R | TACAGAAGGAGATGTCAAAAATCAAAAAAAGCACCGACTCGGTGCCAC |
| pgsCL-F | AAAAAAGCACCGACTCGGTGCCACTGATTTTTGACATCTCCTTCTGTA |
| pgsCL-R | TTGAAAGGTTAATTGTTTTTTCATTTTTCGTACAGCCGATTCAAAATC |
| pgsCR-F | AGGTGTTTTTTTATTACCCTCGAGGCTCCGGCATCTGACATTGCTCTTGATTT |
| pgsCR-R | TGAATCGGCTGTACGAAAAATGAAAAAACAATTAACCTTTCAA |
| sgpgsA-F | AAAAAGGAGCGATTTAGTCGACAGATGCCGTAAAAGTATTGAGTTTTAGAGCTAGAAATAGCAAGT |
| sgpgsA-R | GGCACGGCAGTTCATGATAACAATAAAAAAGCACCGACTCGGTGCCAC |
| pgsAL-F | GTGGCACCGAGTCGGTGCTTTTTTATTGTTATCATGAACTGCCGTGCC |
| pgsAL-R | TTAATGATTTTCATTTGTTTTTCACGTTTGACACACCTTACATTAGAT |
| pgsAR-F | ATCTAATGTAAGGTGTGTCAAACGTGAAAAACAAATGAAAATCATTAA |
| pgsAR-R | AGGTGTTTTTTTATTACCCTCGAGATCTTTTGTAATCCGCTTTGCGC |
| sgargF-F | AAAAGGAGCGATTTAGTCGACTTGACGATACAAGAAATAAAGTTTTAGAGCTAGAAATA |
| sgargF-R | GTCGCCTGAATGGACGCCTGCCCGAAAAAAGCACCGACTCGGTGCCACTTTT |
| argFL-F | GTGGCACCGAGTCGGTGCTTTTTTGTGGCACCGAGTCGGTGCTTTTTT |
| argFL-R | AGCTTTCAGGAGCGCTTTTTGCGCGCCGTACAAATGGGTTAATGTGTG |
| argFR-L | CACACATTAACCCATTTGTACGGCGCGCAAAAAGCGCTCCTGAAAGCT |
| argFR-R | CAGCTTCGGGCTTTGGTCGACTCCGCCTCCTTTTACTCCTAGTC |
| sgargI-F | AAAAGGAGCGATTTAGTCGACAATCTGAACTCTGTGCTCTCGTTTTAGAGCTAGAAATA |
| sgargI-R | CCACTCCCGCCTGCTTCCACGGAATAAAAAAGCACCGACTCGGTGCCACT |
| argIL-F | AGTGGCACCGAGTCGGTGCTTTTTTATTCCGTGGAAGCAGGCGGGAGTGG |
| argIL-R | ACAAGGACTCCACCAGCTCAACCGCTAACCGTTCAATGAGATGCGCGTAG |
| argIR-F | CTACGCGCATCTCATTGAACGGTTAGCGGTTGAGCTGGTGGAGTCCTTGT |
| argIR-R | CAGCTTCGGGCTTTGGTCGACGATCGCCTGCTTTGGCGG |
| argFout-F | GCAAATTTCAGAACTGCTTGAAAACGAAGCGGGCA |
| argFout-R | CACTTTGTCAGCGGGGATTCAAGCTTTTGGCTCGA |
| argIout-F | AAGTGAAAATCCCGAGAAAACGATTCCGCGCTCTATCCGG |
| argIout-R | TTATCGAGAGCCGTTTCAAACTCGACTCGCGCCTCCTCCA |
| Sgprob-F | AAAAGGAGCGATTTAGTCGACGTACAGAAATGCGTATGCCAGTTTTAGAGCTAGAAATA |
| Sgprob-R | AATCTCATACTGCTCCAGAAGCTCAAAAAAAGCACCGACTCGGTGCCACT |
| probL-F | AGTGGCACCGAGTCGGTGCTTTTTTTGAGCTTCTGGAGCAGTATGAGATT |
| probL-R | CAAATAAATTCACCCAATCATTTCTGAGCGAGCTGCTCCCGATTTTCACA |
| probR-F | TGTGAAAATCGGGAGCAGCTCGCTCAGAAATGATTGGGTGAATTTATTTG |
| probR-R | CAGCTTCGGGCTTTGGTCGACGGAAGGCCGCTGTGTTTTG |
| speFL-F | GGTTTCCCTCTAGATAGCGCATGCTGAATTCAAGCAGGAGGAAATCACAAAACAGATTC |
| speFL-R | GCTCTTTGATGACTCTGATCATGTTAAGGTGCTTTTTCAGTCCGCTGTACAGGGGT |
| speFR-F | ACCCCTGTACAGCGGACTGAAAAAGCACCTTAACATGATCAGAGTCATCAAAGAGC |
| speFR-R | TGTATACATACTTTAAAAATCTCGAGGTAAACGGTCAATTTTTGAAAAAAGATG |
| probOUT-F | ACCATGAAGAGGCGAAAGAAGTGGTTGAACCGCTG |
| probOUT-R | GAAGCTCCACCCCTTTTTCTGTCAGCTGATTCAGC |
| speFOUT-F | CCACTTTCAGATCGGATTATGGGACACCCATATGTTTGTATGGTT |
| speFOUT-R | AAAACCGATCGCATTGTTAACGATAGATGATGCACCTGATCAGCA |
| ArgA-F | TGTGCCACCTAAAAAGGAGCGATTTAACTAGTATGATTCAGCTGAGTGAGGAAATCAC |
| ArgA-R | AAAAACAATGGTTTTCCTCATATGTAAATCGCTCCTTTTTATGTGCGATAGCTCGCATT |
| ArgB-F | AATGCGAGCTATCGCACATAAAAAGGAGCGATTTACATATGAGGAAAACCATTGTTTTT |
| ArgB-R | GCCTATAATACCTATTTTCAAATGTAAATCGCTCCTTTTCATGACACGCTCTCCTTTTC |
| ArgC-F | GAAAAGGAGAGCGTGTCATGAAAAGGAGCGATTTACATTTGAAAATAGGTATTATAGGC |
| ArgC-R | CAGCTTGGAGGTGTTTTTTTATTACCCTCGAGCTATGGATAGACTGGCGTCATCGTGA |
| ArgD-F | TGTGCCACCTAAAAAGGAGCGATTTAGGATCCATGAGCAGCTTATTTCAAACCTACAG |
| ArgD-R | CAGCATGTCAAGCTGATTCATATGTAAATCGCTCCTTTTTATCGTTTCACTGCGGAATT |
| ArgE-F | AATTCCGCAGTGAAACGATAAAAAGGAGCGATTTACATATGAATCAGCTTGACATGCTG |
| ArgE-R | AGCTTGGAGGTGTTTTTTTATTACCGCGGCCGCTCACATCGGGCAATCTTTTTTGCGG |
| ArgA-RBS-R | CTCTTTTACATTTTGAAAAAAATGTAAATCCCTCCTTTTTATGTGCGATAGCTCGCATT |
| ArgB-RBS-F | AATGCGAGCTATCGCACATAAAAAGGAGGGATTTACATTTTTTTCAAAATGTAAAAGAG |
| ArgB-RBS-R | GCCTATAATACCTATTTTCAAATGTAAATCCCTCCTTTTCATGACACGCTCTCCTTTTC |
| ArgC-RBS-F | GAAAAGGAGAGCGTGTCATGAAAAGGAGGGATTTACATTTGAAAATAGGTATTATAGGC |
| ArgC-RBS-R | GGTTTGAAATAAGCTGCTCATATGTAAATCCCTCCTTTCTATGGATAGACTGGCGTCAT |
| ArgD-RBS-F | ATGACGCCAGTCTATCCATAGAAAGGAGGGATTTACATATGAGCAGCTTATTTCAAACC |
| ArgD-RBS-R | CAGCATGTCAAGCTGATTCATATGTAAATCCCTCCTTTTTATCGTTTCACTGCGGAATT |
| BA.lysE-F | TGCCACCTAAAAAGGAGCGATTTACTCGAGATGAACATCTTTTTAAGCTACATC |
| BA.lysE-R | TTGCTTTCGAGGTGAATTTCGACCGTCGACTTATGAGATGAGCATCTGAATCCC |
| Cg.lysE-F | TGCCACCTAAAAAGGAGCGATTTACTCGAGATGGTGATCATGGAAATCTTCATT |
| Cg.lysE-R | TTGCTTTCGAGGTGAATTTCGACCGTCGACCTAACCCATCAACATCAGTTTGAT |
| E.coli.lysE-F | TGCCACCTAAAAAGGAGCGATTTACTCGAGATGGAATATTCAACATTATTATCA |
| E.coli.lysE-R | TTGCTTTCGAGGTGAATTTCGACCGTCGACTCACGTAAAAATAAAATAAATACC |
| BS.lysE-F | TGCCACCTAAAAAGGAGCGATTTACTCGAGGTGATTTTGACATCCATTACGCCT |
| BS.lysE-R | TTGCTTTCGAGGTGAATTTCGACCGTCGACCTACAAATAAATACTGACGGCAGA |
